# Individualized volume‐corrected maximum flow rate correlates with outcome from bladder outlet surgery in men with lower urinary tract symptoms

**DOI:** 10.1111/iju.13099

**Published:** 2016-05-15

**Authors:** Alison Bray, Chris Harding, Robert Pickard, Michael Drinnan

**Affiliations:** ^1^ Regional Medical Physics DepartmentNewcastle Upon Tyne Hospitals NHS Foundation TrustNewcastle Upon TyneUK; ^2^ Institute of Cellular MedicineNewcastle UniversityNewcastle Upon TyneUK; ^3^ Urology DepartmentNewcastle Upon Tyne Hospitals NHS Foundation TrustNewcastle Upon TyneUK

**Keywords:** bladder outlet obstruction, home monitoring, nomograms, transurethral resection of prostate, uroflowmetry

## Abstract

**Objectives:**

To develop a per‐patient volume correction for maximum flow rate using multiple home uroflowmetry, and to carry out a pilot study to determine the most prognostically useful volume at which to evaluate this measurement and estimate its relationship with outcome from disobstructive bladder outlet surgery.

**Methods:**

A total of 30 men carried out home uroflowmetry using a portable device and completed symptom scores before surgery. This was repeated at least 4 months after surgery. For each man's presurgery flow data, voided volume was plotted against maximum flow rate, and a line of best fit with logarithmic form calculated. This allowed maximum flow rate to be corrected for any volume. Percentage reduction in symptom score and increase in mean maximum flow rate were correlated with volume‐corrected maximum flow rates.

**Results:**

Corrected maximum flow rate at all volumes showed the expected negative correlation with both outcome measures. A statistically significant correlation occurred for volumes >190 mL, with the best performance at volumes >300 mL.

**Conclusions:**

We have devised a novel method allowing estimation of maximum flow rate at any volume, which is a step forward for non‐invasive diagnostics. We found this volume‐corrected maximum flow rate to correlate significantly with treatment outcome at sufficiently high volumes.

**Abbreviations & Acronyms**
BOO: bladder outlet obstruction
DO: detrusor overactivity
IPSS: International Prostate Symptom Score
LUTS: lower urinary tract symptoms
*N*_voids_: number of voids
PVR: post‐void residual
*Q*_max_: maximum flow rate
QOL: quality of life
*V*_blad_: bladder volume
*V*_void_: voided volume

## Introduction

The decision to carry out surgery for BOO in men with LUTS is guided by uroflowmetry. Typically a one‐off measurement of *Q*_max_ is obtained using office‐based uroflowmetry. There is evidence that when multiple measurements of *Q*_max_ are made for an individual, either the highest^1^ or average^2^ *Q*_max_ improves diagnostic accuracy for BOO.

Ideally, in order to control for dependency on *V*_blad_, *Q*_max_ should be measured at a specific volume, but this is impractical. Clinical guidelines recommend that the *V*_void_ should be at least 150 mL, but this is not always feasible for men with habitual low *V*_void_.^3^, ^4^

Nomograms have been developed enabling *Q*_max_ to be evaluated in the context of *V*_blad_ or *V*_void_, using relationships derived from large groups.^5^, ^6^ However, the relationship between *Q*_max_ and volume varies between individuals.^7^ These nomograms do not necessarily improve diagnostic accuracy, because a larger volume might not actually mean an increased *Q*_max_ in a particular individual.

We aimed to develop a per‐patient correction for flow rate using multiple measurements of *Q*_max_ and *V*_void_ from home uroflowmetry, and carry out a pilot study to determine the most prognostically useful volume at which to evaluate this derived measurement of *Q*_max_ and estimate its relationship with outcome from surgery for BOO.

## Methods

All men scheduled for bladder outlet surgery for LUTS within the Freeman Hospital, Newcastle Upon Tyne, UK, were eligible for invitation into the study. Exclusion criteria were the presence of an indwelling urinary catheter or need for intermittent self‐catheterization. After research approvals and written consent, each man was given a home uroflowmeter and asked to record as many voids as possible for 1 week. They also completed an IPSS questionnaire. This assessment was repeated at least 4 months after surgery. The decision to carry out surgery was made before study enrollment.

The objective outcome from surgery was defined as the increase in mean *Q*_max_ measured by home uroflowmetry. Symptom outcome from surgery was defined as the percentage reduction in total IPSS score.^8^, ^9^

**Figure 1:**
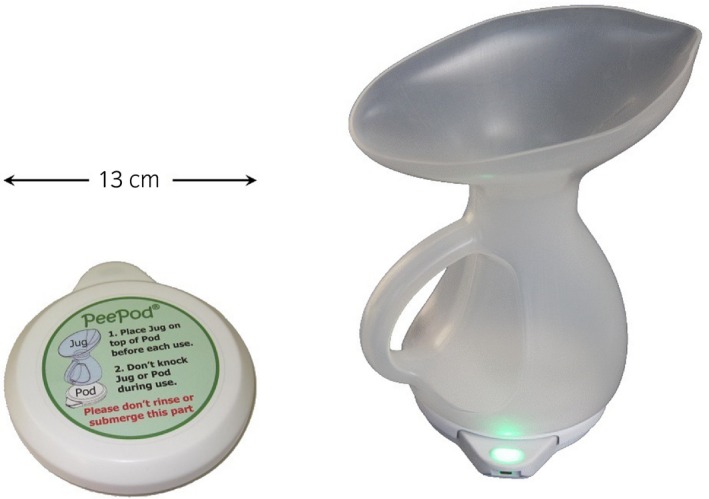
The device used for home uroflowmetry: the measurement base unit (left) and the jug on top of the base unit ready for use (right).

The home flowmeter (Fig. 1) was developed in the Regional Medical Physics Department, Newcastle Upon Tyne Hospitals NHS Foundation Trust. It consists of a jug that is placed on a measurement base unit before voiding into the jug. Volume and flow rate are measured by a weight transducer. It is intended for unsupervised use by patients, and is designed for ease of use, with no controls. It records all voids onto inbuilt memory for up to 2 weeks, after which data are downloaded to a computer. Software obtains *Q*_max_, *V*_void_, date, time, duration and a flow trace for each void. The accuracy of its volume and flow rate measurements is within ±5% relative to full scale (1000 mL and 50 mL/s, respectively), as recommended by the current clinical guideline at the time.^10^ Each void was checked visually to verify, and if necessary correct, automated calculation of void start, void end, *V*_void_ and *Q*_max_.

Urine flow rate depends on *V*_blad_.^11^ Hence Siroky *et al*. developed nomograms relating flow rate to *V*_blad_ according to a polynomial relationship. The home flowmeter measures *V*_void_, requiring assessment of PVR for *V*_blad_ to be known.^12^ At present, there are no tools available for patients to measure their own PVR at home after each void. We therefore used *V*_void_ as a surrogate for *V*_blad_. Using measurements of *Q*_max_ and *V*_void_ from presurgery home uroflowmetry, we planned to calculate an equation of best fit for each man in order to predict *Q*_max_ from *V*_void_. This required selection of the form these equations would take. The Liverpool nomograms are the most widely cited nomograms relating *Q*_max_ to *V*_void_ in men. They were constructed from single measurements of flow obtained from 331 male volunteers with no history of LUTS or bladder‐related surgery.^6^ The nomograms are based on equation (1).

(1) $$\sqrt{(}Q_{\max})=2.37+0.18\times \sqrt{(}V_{\mathrm{void}})-0.014\times \left(\text{age}\right)$$

Two nomograms were chosen with 35 and 60 years for the age term, representing the median ages of the <50 years and ≥50 years cohorts, respectively. We therefore selected a square root relationship for the equations used to predict *Q*_max_ from *V*_void_ before surgery for each man. The equations took the form shown in equation (2).

(2) $$\sqrt{(}Q_{\max})=\left(\beta_{1}\right)\times \sqrt{(}V_{\mathrm{void}})+\left(\beta_{2}\right)$$

**Figure 2:**
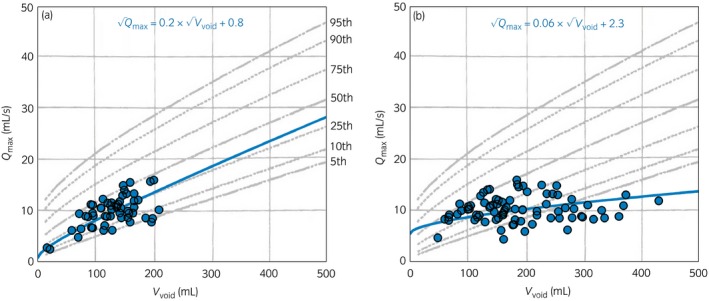
Examples of *Q*_max_ versus *V*_void_ for each void recorded using the home flowmeter for two patients. The lines show the equations $\sqrt{(}Q_{\max})=\left(\beta_{1}\right)\times \sqrt{(}V_{\mathrm{void}})+\left(\beta_{2}\right)$ calculated for each man from his data points. The plots are superimposed over the Liverpool nomogram for maximum flow rate in men aged 50 years and older. The grey lines are the 5–95th centiles from this nomogram, as shown on the first plot.

**Figure 3:**
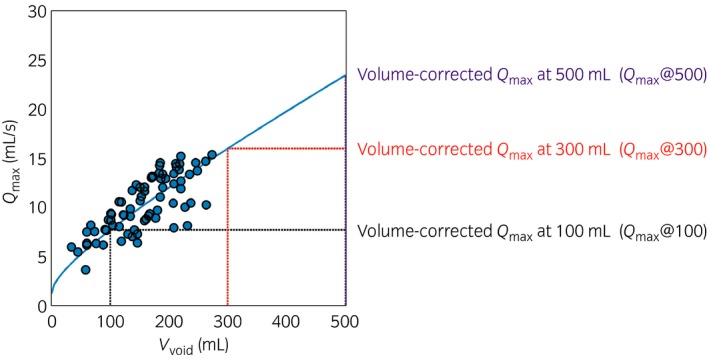
An example of *Q*_max_ versus *V*_void_ data points and their best fit square root relationship for one patient, illustrating the calculation of volume‐corrected *Q*_max_ at 100, 300 and 500 mL.

Coefficients β_1_ and β_2_ were unique for each patient. These parameters were calculated using MATLAB vR2012 software (MathWorks, Natick, MA, USA) to minimize the residual sum of squares between the line described by this equation and the *Q*_max_ values recorded by the home flowmeter. A point at (*V*_void_,*Q*_max_) = (0,0) was included for calculating the coefficients. To illustrate this methodology, Figure 2 shows examples of this equation for two patients, superimposed over the Liverpool nomogram for men aged ≥50 years. Using these equations, a set of “volume‐corrected” *Q*_max_ (*Q*_max_@Vol) values for each individual was calculated for volumes from 0 to 500 mL at 5‐mL intervals, as shown in Figure 3. At each volume, the corrected *Q*_max_ values for the group were correlated with outcome using Spearman's rank, giving a correlation coefficient (rho) and *P*‐value. This allowed calculation of the volume at which the *Q*_max_@Vol values performed best in terms of predicting outcome in the study population.

For this feasibility study, we considered that results from a group of 30 men would be sufficient to determine feasibility of the use of home uroflowmetry and analyses in this context, and to estimate the predictive value for treatment outcome to power follow on studies.^12^ Statistical analyses were carried out using MATLAB vR2012 software.

## Results

We recruited 33 men between January and September 2012. One man withdrew after the first period of recording because of ill health, a second man's prostate procedure was delayed, preventing collection of outcome data and a third participant's operation was postponed after heart surgery. This left 30 datasets available for analysis. The surgical procedures carried out for these 30 men, all for relief of BOO, were: diathermy transurethral resections of the prostate (19); holmium laser enucleation of the prostate (7); potassium titanyl phosphate laser vaporisation of the prostate (3); and holmium laser incision of the prostate (1). Table 1 presents the age and home uroflowmetry statistics for each patient. The median (25–75th percentile) age of men who completed the study was 72 years (67–75 years). Although patients were instructed to use the home flowmeter for 1 week, several continued to do so for longer, for up to a maximum of 2 weeks as dictated by the device's operational period. All voids were included for analysis. Patients recorded a mean (SD) of 54 (25) voids before surgery and 54 (21) voids afterwards. The mean (minimum – maximum) number of voids recorded per day was 8 (2–15) before surgery and 6 (2–9) afterwards (*P* = 3 × 10^−4^, paired *t*‐test). Automated and manual calculations of *Q*_max_ were within 1 mL/s for 91% of voids and within 2 mL/s for 95%. For *V*_void_, 92% were within 10 mL and 96% within 20 mL. Manual verification and, if necessary, correction took between 2 and 15 min per study, depending on the number of voids recorded and corrections required.

**Table 1:** Age and, from pre‐surgery home uroflowmetry, number of voids (*N*_voids_), mean (SD) *V*_void_ and mean (SD) *Q*_max_ for each man

No.	Age (years)	*N*_voids_	Mean (SD) *V*_void_ (mL)	Mean (SD) *Q*_max_ (mL/s)	No.	Age	*N*_voids_	Mean (SD) *V*_void_ (mL)	Mean (SD) *Q*_max_ (mL/s)
1	79	86	104 (49)	6.3 (2)	16	68	34	225 (65)	13.7 (2.5)
2	71	66	142 (64)	9.4 (2.7)	17	70	79	176 (102)	9.5 (2.9)
3	62	46	240 (134)	13.4 (3.6)	18	73	46	184 (86)	10.9 (1.3)
4	86	63	92 (46)	4.1 (1.1)	19	67	34	310 (166)	16.3 (5.2)
5	76	55	130 (42)	9.8 (2.9)	20	75	81	188 (82)	10.3 (2.5)
6	70	64	114 (50)	6.8 (2.1)	21	65	25	102 (44)	3 (0.7)
7	55	38	225 (97)	6.6 (1.2)	22	75	66	106 (31)	6.2 (2.1)
8	83	60	110 (68)	8.7 (2.7)	23	68	14	252 (136)	7.4 (2.4)
9	67	29	337 (135)	12.1 (3.9)	24	63	48	213 (95)	13.8 (3.2)
10	74	42	141 (63)	11.6 (3.8)	25	81	76	159 (58)	10.3 (2.8)
11	73	33	249 (108)	12.3 (2.5)	26	73	60	104 (49)	7.8 (2.5)
12	68	54	214 (67)	5.8 (1.5)	27	68	44	203 (68)	6.9 (1.6)
13	84	59	114 (47)	6.9 (2.1)	28	72	24	119 (33)	5.4 (1.5)
14	64	62	229 (94)	11.2 (1.4)	29	72	64	200 (85)	9.7 (1.8)
15	89	146	102 (68)	3.9 (1.7)	30	62	31	152 (61)	6 (1.8)

Table 2 compares pre‐ and postsurgery values for mean *Q*_max_, mean *V*_void_, total IPSS score, IPSS QOL score, IPSS voiding subscore (sum of IPSS intermittency, weak stream and straining scores) and IPSS storage subscore (sum of IPSS frequency, urgency and nocturia). A total of 24 patients (80%) experienced a significant increase in *Q*_max_ after surgery, and 16 (53%) experienced a significant increase in *V*_void_ (*P* < 0.05, multiple measurements in each man compared before and after surgery using the independent *t*‐test). The median (25–75th percentile) length of time between surgery and follow up was 170 days (156–213 days). There was no correlation between length of time from surgery to follow up and any of the following: increase in mean *Q*_max_, increase in mean *V*_void_ or decrease in total IPSS score (all *P* > 0.05, Spearman's rank).

**Table 2:** Comparison of outcomes before and subsequent to bladder outlet surgery in the study population

	Presurgery	Postsurgery	*P*‐value
Mean *Q*_max_ (mL/s)†	8.9 (3.3)	17.8 (8.2)	2 × 10^−6^
Mean *V*_void_ (mL)†	175 (66)	220 (71)	1 × 10^−4^
IPSS total‡	21 (16–24)	7.5 (3–10)	2 × 10^−6^
IPSS QOL‡	5 (4–6)	2 (0–3)	3 × 10^−6^
IPSS voiding‡	8 (6–10)	1 (0–3.5)	3 × 10^−6^
IPSS storage‡	9.5 (8–11)	5 (3–7)	5 × 10^−6^

Total *n* = 30. †Mean (SD) values and *P*‐value of the comparison according to the paired *t*‐test. ‡Median (25–75th percentile) values and *P*‐value of the comparison according to the Wilcoxon signed rank test.

**Figure 4:**
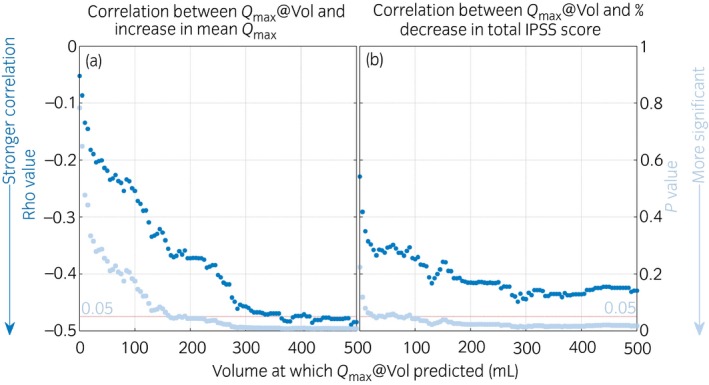
Variation in rho (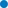) and *P*‐ (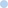) values with volume for the correlation between *Q*_max_@Vol and (a) objective surgical outcome (increase in mean *Q*_max_ from the home flowmeter) and (b) subjective surgical outcome (percentage decrease in total IPSS score).

**Figure 5:**
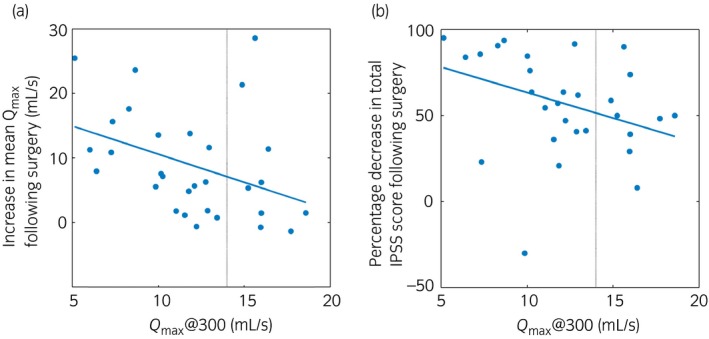
Volume‐corrected *Q*_max_ at 300 mL versus (a) increase in mean *Q*_max_ from home uroflowmetry and (b) percentage decrease in total IPSS score after surgery, with linear best‐fit relationships. The vertical dotted lines show *Q*_max_@300 = 14 mL/s.

For every patient, *Q*_max_@Vol was calculated at 5‐mL intervals from 0 to 500 mL from presurgery home uroflowmetry data. At all volumes, *Q*_max_@Vol for the group showed the expected negative correlation (ρ < 0) with both outcome measures, showing that men with lower presurgery flow rates tended to experience better outcomes. *Q*_max_@Vol values for volumes above 190 mL correlated significantly (*P* < 0.05) with both objective and symptom outcome. Figure 4 shows how rho and *P*‐values changed depending on the volume at which *Q*_max_@Vol was corrected. For objective outcome, correlation improved with increasing volume, plateauing at 300 mL, where rho was less than −0.46 (95% confidence interval −0.12 to −0.70) and *P* < 0.01 (moderate correlation; Fig. 4a). A similar, but less pronounced, pattern was observed for the correlation between *Q*_max_@Vol and symptom outcome (Fig. 4b). Figure 5 shows volume‐corrected *Q*_max_ at 300 mL (*Q*_max_@300) versus (i) increase in mean *Q*_max_ from home uroflowmetry and (ii) percentage decrease in total IPSS score after surgery, both with linear least squares line of best fit.

Given that the flow rate is reported to the nearest 1 mL/s, it would be sensible to record enough voids such that inclusion of additional voids does not change *Q*_max_@300 by more than ±0.5 mL/s.^10^ This was calculated for each individual, and the highest void number at which this occurred was 21.

We defined a man's *Q*_max_ − *V*_void_ relationship as one that “fit” the Liverpool nomogram if it intersected fewer than two of the Liverpool centile lines for *V*_void_ > 100 mL. Accordingly, 14 (47%) did fit the Liverpool nomogram (e.g. Fig. 2a) and 16 (53%) did not (e.g. Fig. 2b).

## Discussion

Use of a single volume‐corrected *Q*_max_ or nomogram categorization of (*V*_void_,*Q*_max_) is used as a method to decide whether an individual's *Q*_max_ is abnormally low, in order to determine the likelihood of BOO and guide clinical management. Methods reported previously have derived a relationship between volume and *Q*_max_ using a single or small number of voids from each individual in a large group of asymptomatic men. Either *V*_void_, measured by the flowmeter, or *V*_blad_, determined by summing *V*_void_ and PVR, have been used. A single flow measurement from a man with LUTS is then evaluated in this context. This approach risks poor diagnostic and prognostic accuracy if the patient under assessment does not show the assumed relationship between *Q*_max_ and *V*_void_. Sonke *et al*. found this relationship to differ considerably between individuals, with one‐third having a negative relationship (decrease in *Q*_max_ with increasing *V*_void_).^7^ Only a large number of measurements obtained from an individual allow their true relationship to be determined with statistical confidence. This is made possible by asking patients to carry out uroflowmetry on multiple occasions at home, or elsewhere, during their day‐to‐day life.

A group of men with LUTS will include men with BOO, men with DO, men with both BOO and DO, and men with neither. Bladder outlet surgery is thought to achieve better outcomes in men with BOO than those without, and symptoms associated with DO in men with BOO have been observed to improve after disobstructive surgery.^13^, ^14^ Ideal candidates for surgery are therefore men with BOO, with or without coincident DO. A one‐off measurement of flow with low *V*_void_ and low *Q*_max_ cannot differentiate a man with BOO from one with pure DO, in whom disobstructive surgery is less likely to give benefit. The method presented in this study allows estimation of *Q*_max_ at any volume by extrapolation of serial voids, which might be low volume. This means that there is in effect no lower volume limit for flow data acquired in this way, which is a step forward for non‐invasive diagnostics.

The present study, using this approach, found that a volume‐corrected *Q*_max_ calculated for each man from paired measurements of *Q*_max_ and *V*_void_ correlates well with outcome from disobstructive surgery, suggesting that it might be valuable in predicting who would benefit from surgical treatment. For objective outcome, better and statistically significant correlation was measured for volume‐corrected *Q*_max_ at volumes of 300 mL and above, which might reflect the average volume above which the detrusor must be stretched to generate its greatest contractile force. Thus, the present results suggest that the optimum volume is at least twice the 150 mL recommended minimum *V*_void_ for a valid office‐based flow test.^3^, ^4^

Fixing a point of origin at (*V*_void_,*Q*_max_) = (0,0) allows mathematical calculation of a square root relationship from just one void, but an estimate based on a few voids is inaccurate. With each additional recorded void, *Q*_max_@300 tends towards its true value. In the present study, for no patient was there any practical improvement in accuracy beyond 21 voids. For future larger scale independent validation studies, we therefore recommend that at least 21 voids are recorded per man for reliable calculation of *Q*_max_@300. All but one of our participants recorded at least this number (Table 1), despite no minimum being requested.

The process of fitting an equation to predict *Q*_max_ from *V*_void_ was carried out using MATLAB vR2012 software. In future, these calculations could be built into the home uroflowmetry analysis software for presentation alongside other summary statistics from the study.

Previously, subjective outcome from surgery has been defined by percentage improvement in IPSS, with <50% classed as “poor” outcome and ≥50% classed as “good.”^8^, ^9^ Thus, from Figure 5b, one may apply a threshold of 14 mL/s to *Q*_max_@300, because at this value the line of best fit between *Q*_max_@300 and percentage decrease in IPSS after surgery equals 50%. A threshold approach like this is often favored for research purposes, allowing cases to be grouped and compared. However, it would be more valuable clinically to use the relationship between *Q*_max_@300 and outcome to inform a man of the improvements in symptoms and flow rate expected on average for a man with similar presurgery flow volume characteristics. This could inform the decision of whether or not to undergo surgery.

Few previous studies have correlated presurgery uroflowmetry parameters with outcome. Oh *et al*. measured in 134 men the correlations between pretransurethral resections of the prostate BOO index both pre‐surgery *Q*_max_ from single clinic uroflowmetry (ρ = 0.26, *P* = 0.002) and improvement in IPSS storage subscore (ρ = 0.19, *P* = 0.032).^15^ However, this was a different population of patients, preselected according to BOO index, and using Pearson's analysis, which produces weaker correlation for non‐linear relationships.

As a feasibility study, this suffered from several limitations. Different surgical procedures were carried out, although all were carried out to relieve BOO. There was variation in the diagnostic workup and treatment history of participants, and subgroup analyses would have lacked statistical power. All patients were deemed by their urologist to be suitable for disobstructive bladder outlet surgery, although pressure‐flow studies to confirm BOO were carried out only in men with higher operative risk (fewer than half). There was variation in time between surgery and follow up for logistical reasons, but most men were reinvestigated at 4–6 months, and no difference in outcomes was observed according to time to follow up. Despite these limitations, a significant relationship between *Q*_max_@Vol and surgical outcome was shown. For reasons described earlier, a volume‐corrected *Q*_max_ should perform better than a single office‐based measurement in terms of predicting treatment outcome. However, the lack of a direct comparison in the present study, because of the unavailability of these results in a number of participants, was another limitation.

We now plan to investigate the diagnostic and prognostic value of *Q*_max_@Vol in larger groups of men with LUTS. The most appropriate function to predict *Q*_max_ from *V*_void_ for a cohort (in this case a square root function) is not necessarily the most appropriate for each man (this is a recognized phenomenon: “Simpson's paradox” describes the general situation where a group relationship is non‐existent or reversed in the individual^16^). There is therefore scope for further work to determine the most appropriate mathematical function to predict *Q*_max_ from *V*_void_ in an individual. Indeed, deviation from a normal pattern could indicate urological abnormality, and classification of *Q*_max_ versus *V*_void_ patterns related to different urological diseases would be of great interest. A large normative dataset and method of quantifying whether an individual fits a normal pattern are required. It would be interesting to investigate the effect of the number of voids recorded and voided volumes on the clinical value of *Q*_max_@300.

In conclusion, we have devised a novel method for providing, for each individual, an estimate of *Q*_max_ at specific *V*_void_, using multiple home uroflowmetry. We found in a limited patient sample that this volume‐corrected *Q*_max_ at volumes above 190 mL correlated well with treatment outcome, with optimum volumes being 300 mL and above. Early data suggest a threshold of 14 mL/s for *Q*_max_@300. These results can be used to power larger studies of the diagnostic and predictive value of volume‐corrected *Q*_max_ in men with LUTS.

## Conflict of interest

Two of the authors are co‐inventors of the home flowmeter, which has been licenced to Medical Measurement Systems Bv as “Flowtaker.” Their employer will receive royalties from sales of this device.
